# The predictive role of modified stress hyperglycemia rate in predicting early pneumonia after isolated coronary bypass surgery in patients with diabetes mellitus

**DOI:** 10.17305/bb.2024.10330

**Published:** 2024-08-21

**Authors:** Ahmet Kağan As, Mesut Engin

**Affiliations:** 1 Department of Cardiovascular Surgery, University of Health Sciences, Bursa Yuksek Ihtisas Training and Research Hospital, Bursa, Türkiye

**Keywords:** Coronary artery bypass graft (CABG), postoperative term, pneumonia, risk factor

## Abstract

Postoperative pneumonia (PP) is one of the most serious complications following coronary artery bypass graft (CABG) surgery. The recently developed admission blood glucose (ABG)/estimated average glucose (eAG) ratio has been identified as a prognostic marker in cardiovascular diseases. This study aimed to investigate the predictive role of the modified ABG/eAG (mABG/eAG) ratio in the development of pneumonia during the early postoperative period in diabetic patients undergoing CABG surgery. In this single-center study, diabetic patients who underwent isolated coronary bypass surgery at the Training and Research Hospital between January 1, 2018 and January 1, 2023 were included. Patients who did not develop PP were assigned to the control group, while those who developed PP were assigned to the PP group. A total of 549 patients were included in the study, 478 patients in the control group (median age ═ 58 years [range 35–81]) and 71 patients in the PP group (median age ═ 63 years [37–86]). In the multivariate analysis, the use of packed blood products (odds ratio [OR] ═ 1.685, 95% confidence interval [CI] 1.453–1.892; *P* ═ 0.027), mABG/eAG ratio (OR ═ 1.659, 95% CI 1.190–2.397; *P* ═ 0.019), and re-intubation (OR ═ 1.829, 95% CI 1.656–1.945; *P* ═ 0.034) were identified as independent predictors for the development of PP. Our findings demonstrate that the mABG/eAG ratio is an independent predictor of PP in diabetic patients undergoing CABG surgery. Based on our results, high-risk patients can be identified by calculating the mABG/eAG ratio.

## Introduction

Coronary artery bypass graft (CABG) surgery is a procedure performed with high success rates today; however, serious complications that can occur following these operations remain significant concerns [1]. Postoperative pneumonia (PP) is among the most severe complications [2]. This condition is associated with prolonged hospital stays and an increased risk of morbidity and mortality. According to the literature, the rate of PP ranges from 5% to 21%, with mortality rates varying between 6.2% and 28% in these cases [3–5]. Various inflammatory parameters obtained from routine blood tests in cardiovascular surgery have been the subject of research for their prognostic value. These parameters have played a role both in the diagnostic approach and as prognostic markers [6, 7]. Hyperglycemia is one of the stress-related factors that requires close monitoring during and after major surgical procedures, particularly in cardiac surgery. Numerous publications have reported that hyperglycemia is associated with a high risk of morbidity and mortality in these cases [8–14]. Beyond hyperglycemia, the recently developed admission blood glucose (ABG)/estimated average glucose (eAG) ratio has emerged as a prognostic marker in cardiovascular diseases [15, 16]. One study demonstrated a relationship between the ABG/eAG ratio and the development of pneumonia in stroke patients [17]. Another study investigating the predictive power of this ratio found that the ABG/eAG ratio was associated with mortality and poor outcomes in patients with COVID-19 [18].

In the current study, we aimed to investigate the predictive role of the modified ABG/eAG (mABG/eAG) ratio, created by modifying blood glucose data, in the development of pneumonia during the early postoperative period following CABG surgery in diabetic patients.

## Materials and methods

This study was designed as a single-center investigation, including diabetic patients who underwent isolated coronary bypass surgery with cardiopulmonary bypass (CPB) at the Bursa Yüksek İhtisas Training and Research Hospital between January 1, 2018 and January 1, 2023. Patients who underwent re-operations, emergency cases, those requiring additional cardiac interventions other than isolated coronary bypass surgery, those with a known history of lung disease, those with chronic renal failure, a history of previous pneumonia, and non-diabetic patients were all excluded from the study. After applying the exclusion criteria, a total of 549 consecutive patients were included. The demographic characteristics of all patients, preoperative blood values, operative data, and blood glucose measurements during surgery were recorded. Patients who did not develop PP were assigned to the control group, while those who developed PP were assigned to the PP group.

### Operative management

All procedures were performed through median sternotomy and under CPB. Pedicled left internal thoracic arteries and saphenous vein grafts were harvested in all patients. Radial artery or right internal thoracic artery grafts were not used in the patients included in the study. Aorto-venous two-stage cannulation was performed to establish CPB following heparinization. To achieve cardiac arrest, a cross-clamp was applied to the ascending aorta, and cold antegrade cardioplegia with high potassium was administered (PLEGISOL^®^ | Pfizer). Blood cardioplegia was administered every 15–20 min to maintain cardiac arrest. CPB was maintained using a roller pump equipped with a membrane oxygenator and arterial line filter (Maquet, Getinge Group, Germany). The pump flow rates were set between 2 and 2.4 L/min/m^2^, and moderate hypothermia (32 ^∘^C) was utilized. Arterial blood gas was evaluated every 20–30 min; immediately before removing the cross-clamp, 500 mL of warm blood cardioplegia were administered. Upon completion of the surgery, the patients were transferred to the cardiovascular intensive care unit. Intra-aortic balloon pump (IABP) support was provided to patients exhibiting low cardiac output, visibly deteriorating cardiac function, and resistant cardiac arrhythmias. All patients received standard postoperative care and were evaluated hourly for extubation readiness. Hemodynamic stabilization (absence of severe cardiac rhythm problems, no need for high-dose vasoactive inotropic support, urine output >0.5 mL/kg/h) was followed by extubation as soon as feasible.

### Calculation of mABG/eAG

Blood parameters were obtained from peripheral venous samples of all patients during their hospitalization. The ABG/eAG ratio, also known as the stress hyperglycemia ratio, is calculated by comparing the patient’s hyperglycemia response to stress with the estimated glucose value [19]. In our study, the ABG value was established as the average of blood glucose levels obtained from all analyses—from the blood gas analysis before anesthesia induction in the operating room to the first blood gas analysis after transfer to the intensive care unit at the end of the operation. After this modification, the mABG/eAG value was calculated using the following formula:

mABG ═ Blood glucose levels (Before induction of anaesthesia + after induction + during the surgery + on admission to intensive care unit) (mg/dL)/*n*.

The *n* represents the number of blood glucose tests conducted in the perioperative period (this number ranged from 6 to 10 in our study group).

The eAG value was calculated using the following formula:

eAG ═ (28.7 × glycosylated hemoglobin %) −46.7.

### Diagnosis of postoperative pneumonia

In patients with clinically suspected pneumonia, the presence of newly detected infiltration on chest X-ray or an increase in the degree of existing infiltration was considered indicative of pneumonia. Additionally, the diagnosis of pneumonia was confirmed if at least two of the following criteria were met [20]: 1) Fever (>38.5 ^∘^C) or hypothermia (<36.0 ^∘^C); 2) Presence of purulent tracheobronchial secretions or an increase in the quantity of existing secretions; and 3) Leukocytosis (≥12,000/µL) or leukopenia (≤4000/µL). C-reactive protein and procalcitonin levels were also utilized to support the diagnosis.

### Ethical statement

This study was approved by the Clinical Research Ethics Committee of the Bursa Yuksek Ihtisas Training and Research Hospital, as per protocol, on March 22, 2023 (Approval number: 2011-KAEK-25 2023/03-14).

### Statistical analysis

The data were analyzed using SPSS 21.0 (IBM Statistical Package for the Social Sciences, version 21.0, Chicago, IL, USA). The Kolmogorov–Smirnov test and Shapiro–Wilk test were utilized to assess the normality of the data distribution. For data with a normal distribution, Student’s *t*-test was applied, while the Mann–Whitney *U* test was used for data that did not conform to normal distribution. The results were presented as mean ± standard deviation (SD) for normally distributed data or as median (minimum–maximum) for non-normally distributed data. Categorical variables were presented as frequencies and percentages, with the chi-square test used for their analysis. Multivariate binary logistic regression analysis was performed to identify predictors of PP. A *P* value of less than 0.05 was considered statistically significant. Receiver operating characteristic (ROC) curve analysis was conducted to predict in-hospital pneumonia development based on the mABG/eAG value, and the area under the curve (AUC) was calculated. Youden’s J statistic was used to determine the optimal cut-off value for the mABG/eAG ratio.

## Results

A total of 549 patients were included in the study. The diagnosis of pneumonia was made at a mean of 3.4 ± 2.6 days postoperatively. Patients who did not develop PP were included in the control group (*n* ═ 478, median age ═ 58 years [35–81]), while those who developed pneumonia were included in the PP group (*n* ═ 71, median age ═ 63 years [37–86]). The median age of patients was statistically significantly higher in the PP group (*P* < 0.001). The proportion of female patients was 32.6% (*n* ═ 156) in the control group and 36.6% (*n* ═ 26) in the PP group (*P* ═ 0.506). There were no statistically significant differences between the two groups regarding body mass index (BMI), presence of hypertension (HT), smoking, hyperlipidemia (HL), and left ventricular ejection fraction (EF) (Table 1). The preoperative blood values of the patients are shown in Table 1.

**Table 1:** Preoperative characteristics and laboratory variables of the patients

**Variables**	**Control group** **(*n* ═ 478)**	**PP group** **(*n* ═ 71)**	***P* value**
Age, years	58 (35–81)	63 (37–86)	<0.001
Female gender, *n* (%)	156 (32.6)	26 (36.6)	0.506
BMI, kg/m^2^	27.3 (21.9–43)	27.7 (22.1–41)	0.335
Hypertension, *n* (%)	325 (68)	49 (69)	0.863
COPD, *n* (%)	79 (16.5)	20 (28.2)	0.017
Smoking, *n* (%)	98 (20.5)	19 (26.8)	0.230
Hyperlipidemia, *n* (%)	194 (40.6)	35 (49.3)	0.165
OAD use with insulin, *n* (%)	189 (39.5)	30 (42.2)	0.663
Ejection fraction, %	50 (25–65)	50 (30–65)	0.447
White blood cell count, 10^3^/µL	8.8 (4.6–11.2)	9.1 (4.1–10.7)	0.654
Hemoglobin, mg/dL	13.1 (11–16.2)	12.9 (11.4–15.7)	0.076
Platelet count, 10^3^/µL	244.8 (137–440.9)	251.8 (148–390)	0.515
ABG, mg/dL	196 (180–390)	200 (176–420)	0.121
HbA1c, %	7.1 (6.9–11.1)	7.2 (6.7–10.8)	0.439
eAG, mg/dL	159 (149.4–269.8)	165 (146–255.9)	0.081
Creatinine, mg/dL	1 (0.8–1.88)	0.9 (0.7–1.91)	0.418
BUN, mg/dL	12 (10–34)	14 (8–32)	0.196
Albumin, g/dL	4.1 (3.8–5.3)	4.2 (3.6–5.2)	0.289
CRP, mg/L	5.7 (0.7–9)	5.5 (0.6–11)	0.321

Numerical variables were presented as median (range). BMI: Body mass index; COPD: Chronic obstructive pulmonary disease; BUN: Blood urea nitrogen; CRP: C reactive protein; ABG: Admission blood glucose; HbA1c: Hemoglobin A1c; eAG: Estimated average glucose; OAD: Oral antidiabetic drug; PP: Perioperative pneumonia.

The perioperative characteristics of the patients are presented in Table 2. The use of packed blood products, the rate of IABP use, mABG levels, mABG/eAG ratio, extubation times, re-intubation rates, and postoperative atrial fibrillation (PoAF) rates were all statistically significantly higher in the PP group (*P* < 0.001, *P* ═ 0.002, *P* ═ 0.019, *P* < 0.001, *P* ═ 0.012, *P* ═ 0.029, and *P* ═ 0.017, respectively). Additionally, the mortality rate was higher in the PP group (1.8% vs 7%; *P* ═ 0.010).

**Table 2:** Operative and postoperative characteristics of the patients

**Variables**	**Control group (*n* ═ 478)**	**PP group (*n* ═ 71)**	***P* value**
Total perfusion time	92 (55–195)	98 (60–200)	0.116
Cross-clamp time	54 (25–135)	56 (28–166)	0.218
CABG number, *n* (%)	3 (1–6)	3 (1–6)	0.675
Packed blood products (units)	4 (3–9)	7 (4–12)	<0.001
Use of IABP, *n* (%)	39 (8.2)	14 (19.7)	0.002
Inotropic support, *n* (%)	101 (21.1)	20 (28.2)	0.182
^a^Number of BG measurements	7.2 ± 1.9	7.3 ± 1.8	0.769
mABG	185 (108–396)	198 (96–342)	0.019
mABG/eAG	1.12 (0.69–2.38)	1.34 (0.71–2.44)	<0.001
Extubation time, hours	6 (4–12)	8 (4–24)	0.012
Re-intubation, *n* (%)	6 (1.3)	4 (5.6)	0.029
PoAF, *n* (%)	95 (19.9)	23 (32.4)	0.017
Mortality, *n* (%)	9 (1.8)	5 (7)	0.010

^a^Data was presented as mean ± SD, other numerical variables were presented as median (range). CABG: Coronary artery bypass graft; IABP: Intra-aortic balloon pump; mABG: Modified admission blood glucose; eAG: Estimated average glucose; PoAF: Postoperative atrial fibrillation; BG: Blood glucose; PP: Perioperative pneumonia; SD: Standard deviation.

Logistic regression analysis was performed to identify factors influencing the development of PP (Table 3). In the univariate analysis, the development of PP was significantly associated with age > 70 (odds ratio [OR] ═ 1.347, 95% confidence interval [CI] 1.190–1.740; *P* ═ 0.007), chronic obstructive pulmonary disease (COPD) (OR 1.114, 95% CI 1.018–1.668; *P* ═ 0.022), use of packed blood products (OR 0.775, 95% CI 0.535–0.889; *P* < 0.001), use of IABP (OR 0.855, 95% CI 0.537–0.971; *P* ═ 0.006), mABG levels (OR 1.648, 95% CI 1.452–1.856; *P* ═ 0.023), mABG/eAG ratio (OR 2.361, 95% CI 1.796–4.650; *P* < 0.001), extubation time (OR 1.812, 95% CI 1.715–1.994; *P* ═ 0.019), and re-intubation (OR 1.530, 95% CI 1.250–2.194; *P* ═ 0.021). In the multivariate analysis, the use of packed blood products (OR 1.685, 95% CI 1.453–1.892; *P* ═ 0.027), mABG/eAG ratio (OR 1.659, 95% CI 1.190–2.397; *P* ═ 0.019), and re-intubation (OR 1.829, 95% CI 1.656–1.945; *P* ═ 0.034) were identified as independent predictors of PP.

**Table 3:** Logistic regression analysis identifying factors associated with the development of postoperative pneumonia

**Variables**	**Univariate analysis**			**Multivariate analysis**		
	***P***	**Exp(B) odds ratio**	**95% CI** **lower–upper**	***P***	**Exp(B) odds ratio**	**95% CI** **lower–upper**
Age > 70 years	0.007	1.347	1.190–1.740	0.076	1.685	0.944–1.984
COPD	0.022	1.114	1.018–1.668	0.218	1.139	0.845–1.730
Packed blood products	< 0.001	0.775	0.535–0.889	0.027	1.685	1.453–1.892
Use of IABP	0.006	0.855	0.537–0.971	0.138	1.176	0.989–1.296
mABG	0.023	1.648	1.452–1.856	–	–	–
mABG/eAG	< 0.001	2.361	1.796–4.650	0.019	1.659	1.190–2.397
Extubation time, hours	0.019	1.812	1.715–1.994	0.285	1.190	0.796–1.370
Re-intubation	0.021	1.530	1.250–2.194	0.034	1.829	1.656–1.945

COPD: Chronic obstructive pulmonary disease; IABP: Intra-aortic balloon pump; mABG: Modified admission blood glucose; eAG: Estimated average glucose; CI: Confidence interval.

**Figure 1.**
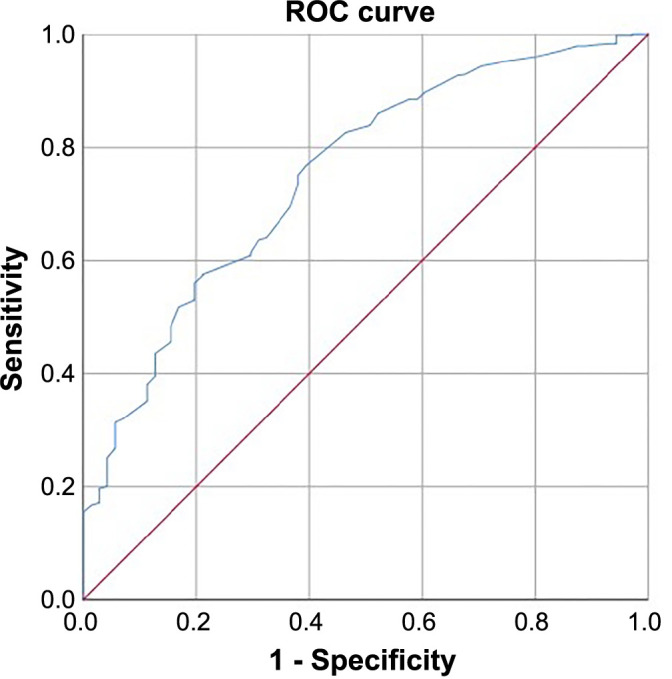
**ROC curve analysis showing the AUC, CI, and cut-off values for mABG/eAG in predicting postoperative in-hospital pneumonia development (cut-off ═ 1.23; AUC ═ 0.750, 95% CI 0.691–0.810; *P* < 0.001, with 75.7% sensitivity and 69.1% specificity).** mABG: Modified admission blood glucose; eAG: Estimated average glucose; ROC: Receiver operating characteristic; AUC: Area under the curve; CI: Confidence interval.

For predicting PP, the cutoff level in the ROC curve analysis was 1.23 for the mABG/eAG ratio (AUC 0.750, 95% CI 0.691–0.810; *P* < 0.001), with a sensitivity of 75.7% and a specificity of 69.1% (Figure 1).

## Discussion

Advancements in cardiac surgery technology have significantly reduced postoperative mortality. However, the incidence of PP remains high, with rates reported to be as much as 21% [21, 22]. Several studies have explored various predictive factors for the risk of PP [23]. In this study, we demonstrated that the mABG/eAG ratio is an independent predictor of pneumonia risk, alongside known risk factors, such as re-intubation and excessive blood product use, following isolated CABG surgery.

The incidence of coronary artery disease is higher among patients with diabetes mellitus, who consequently require CABG more frequently [24]. It has been shown that preoperative hyperglycemia increases the risk of various postoperative complications, and high hemoglobin A1c (HbA1c) levels, an important indicator of long-term blood sugar control, negatively affect outcomes in diabetic patients undergoing CABG [25, 26].

In recent years, the stress hyperglycemia ratio (ABG to eAG ratio), calculated using the blood glucose value and HbA1c at the time of admission, has been widely investigated for its prognostic value in cardiovascular diseases. For instance, in a study involving diabetic patients with ST-segment elevation myocardial infarction, the ABG/eAG ratio was significantly associated with the amount of intracoronary thrombus. The study also found that the predictive power of the ABG/eAG ratio was superior to that of blood glucose levels at admission [27]. Similarly, in non-surgical diabetic patients with heart failure, the ABG/eAG ratio at hospital admission was associated with acute renal injury and major systemic infections [28]. A recent study published in mid-2023 highlighted a significant relationship between a high ABG/eAG ratio and the complexity of coronary artery disease in patients with acute coronary syndrome and diabetes mellitus [29].

Another study evaluated the importance of the ABG/eAG ratio in predicting pneumonia risk following type A acute aortic dissection surgery, a cardiovascular emergency. This retrospective study of 124 patients identified the ABG/eAG ratio, along with prolonged ventilation time, as an independent predictor of PP [30]. In a multicenter, retrospective study involving 1631 diabetic patients, a significant relationship was found between the ABG/eAG ratio and adverse outcomes in patients presenting with pneumonia clinic [31]. Additionally, in a retrospective study of 395 patients hospitalized for COVID-19, the ABG/eAG ratio calculated from admission blood values was associated with worse outcomes and in-hospital mortality [18].

Unlike previous studies, in our research, we derived the ABG value from the average perioperative blood glucose levels, reflecting the operational stress experienced by patients. We recorded this as the mABG value. We demonstrated that the mABG/eAG ratio is an independent predictor of pneumonia risk following isolated CABG surgery. Typically, the ABG/eAG ratio in the literature is calculated using blood glucose levels obtained during acute stress [27–31]. However, our patient group consisted of insulin-dependent diabetic patients who were hospitalized and prepared for elective CABG surgery. We calculated the average blood glucose levels during surgery, based on their response to surgical stress. Therefore, we used the mABG value instead of the standard ABG value in our study.

Additionally, we found that re-intubation and increased blood product use were independent predictors of pneumonia risk. The risk of nosocomial pneumonia increases with repeated intubation. A study by Perrotti et al. [23] revealed that the incidence of pneumonia increased in patients who required re-intubation after elective cardiac surgery. During blood product storage, various immunosuppressive substances are transferred from white blood cells to red blood cells, creating an immunomodulatory state that increases the risk of postoperative infection [32]. A study by Topal and Eren [33] identified increased blood transfusion as an independent predictor of pneumonia risk following cardiac surgeries.

Our study has several limitations. It was a single-center, retrospective study, which presents inherent limitations. Additionally, the study was restricted to patients undergoing isolated coronary bypass surgery, and the relatively small sample size is another constraint. Blood glucose levels in CABG operations performed with CPB may be influenced by many factors. Prolonged ventilation and various postoperative morbidities can also lead to increased blood glucose levels, which is a significant limitation of our study. Multicenter prospective studies with continuous blood glucose monitoring throughout the perioperative period are needed.

## Conclusion

This study introduces a new perspective by modifying the ABG/eAG ratio, which has recently been increasingly used to predict disease development. We demonstrated that our modified mABG/eAG ratio is an independent predictor of pneumonia development in diabetic patients undergoing coronary bypass surgery. Furthermore, when combined with the amount of blood product use, the predictive value was strengthened. Based on our results, high-risk patients can be identified by calculating the mABG/eAG ratio.

## Data Availability

Data supporting this article will be made available by the corresponding author upon reasonable request.
